# PCG-net: feature adaptive deep learning for automated head and neck organs-at-risk segmentation

**DOI:** 10.3389/fonc.2023.1177788

**Published:** 2023-10-20

**Authors:** Shunyao Luan, Changchao Wei, Yi Ding, Xudong Xue, Wei Wei, Xiao Yu, Xiao Wang, Chi Ma, Benpeng Zhu

**Affiliations:** ^1^ School of Integrated Circuit, Wuhan National Laboratory for Optoelectronics, Huazhong University of Science and Technology, Wuhan, China; ^2^ Key Laboratory of Artificial Micro and Nano-structures of Ministry of Education, Center for Theoretical Physics, School of Physics and Technology, Wuhan University, Wuhan, China; ^3^ Department of Radiation Oncology, Hubei Cancer Hospital, TongJi Medical College, Huazhong University of Science and Technology, Wuhan, Hubei, China; ^4^ Department of Radiation Oncology, The First Affiliated Hospital of University of Science and Technology of China, Division of Life Sciences and Medicine, University of Science and Technology of China, Hefei, Anhui, China; ^5^ Department of Radiation Oncology, Rutgers-Cancer Institute of New Jersey, Rutgers-Robert Wood Johnson Medical School, New Brunswick, NJ, United States

**Keywords:** head and neck cancer, radiation therapy, medical image, deep learning, automated segmentation

## Abstract

**Introduction:**

Radiation therapy is a common treatment option for Head and Neck Cancer (HNC), where the accurate segmentation of Head and Neck (HN) Organs-AtRisks (OARs) is critical for effective treatment planning. Manual labeling of HN OARs is time-consuming and subjective. Therefore, deep learning segmentation methods have been widely used. However, it is still a challenging task for HN OARs segmentation due to some small-sized OARs such as optic chiasm and optic nerve.

**Methods:**

To address this challenge, we propose a parallel network architecture called PCG-Net, which incorporates both convolutional neural networks (CNN) and a Gate-Axial-Transformer (GAT) to effectively capture local information and global context. Additionally, we employ a cascade graph module (CGM) to enhance feature fusion through message-passing functions and information aggregation strategies. We conducted extensive experiments to evaluate the effectiveness of PCG-Net and its robustness in three different downstream tasks.

**Results:**

The results show that PCG-Net outperforms other methods, improves the accuracy of HN OARs segmentation, which can potentially improve treatment planning for HNC patients.

**Discussion:**

In summary, the PCG-Net model effectively establishes the dependency between local information and global context and employs CGM to enhance feature fusion for accurate segment HN OARs. The results demonstrate the superiority of PCGNet over other methods, making it a promising approach for HNC treatment planning.

## 1 Introduction

Head and neck cancer (HNC) is the seventh most common cancer worldwide, resulting in an estimated 50,000 deaths in 2018 (1). Radiotherapy is the most commonly prescribed curative treatment option. Evidence showed that it took about 2.7 to 3 hours to delineate a full set of necessary structures in one HNC patient (2), including 0.5 to 1 hour’s organs-at-risk (OARs) delineation. Nowadays, the delineation process is usually performed manually on treatment planning system (TPS). Manual delineation exists inter-variability, which is highly related to knowledge, experience, and preference of the radiation oncologists (3). The OARs auto-segmentation system can save the contouring time from at least half hour to only several minutes. However, the accuracy of commercial auto-segmentation system still needs to be evaluated and improved (4).

Traditional techniques including atlas-based methods (5, 6) and hybrid model-based methods (7, 8) have been used in clinical practice to improve the efficacy and accuracy. The atlas-based process implements segmentation by aligning a fixed set of manually labeled examples with the new images. Hybrid model-based approaches were done by statistical analysis of ground truth contours and imposed prior shape constraints in the segmentation process. These methods may be limited due to large anatomical variations of human organs or local uncertainty of deformable registration (9, 10).

Currently, deep learning represented by deep convolutional neural networks (CNNs) has shown great success in computer science and medical image analysis. There have been many studies which applied CNNs to segment various organs and substructures in radiotherapy for various disease sites and various types of image data (11–16). Given the varying sizes of the OARs) within the head and neck region, we opted to use this particular set of OARs for evaluating the segmentation performance of our deep neural network model. This choice enables a comprehensive assessment of the model’s segmentation abilities across a range of anatomical structures, contributing to a more robust and clinically relevant evaluation. Ibragimov first performed the convolutional neural networks to segment the OARs in head and neck (HN) CT images, and the DSC varied from 37.4% for optic chiasm to 89.5% for mandible (17). Sun et al. developed a first locating then segmentation approach for accurate CT image segmentation of eyes and surrounding organs, which is accurate, efficient, and suitable for clinical use (18). Zhu et al. proposed an end-to-end atlas-free and fully automated deep learning model for anatomy segmentation from HN CT images, which introduced a new encoding scheme, 3D squeeze-and-excitation residual blocks, and combined loss. The experiments showed that compared to the prior state-of-the-art results achieved during the MICCAI 2015 competition, their model exhibited an average increase of 3.3% in the Dice similarity coefficient (19).

However, firstly, traditional deep learning segmentation requires large amounts of annotated datasets, while obtaining the annotated datasets in medical image analysis requires manual layer-by-layer annotation by experienced clinicians (20). Moreover, different institutions have different imaging modalities/protocols and different annotation approaches. Therefore, it is extremely hard to achieve cross-institution tasks by only using supervised training strategies. Secondly, OARs contain regions of variable sizes, including some OARs with very small sizes, such as optic chiasm and optic nerves. Accurately segmenting these small OARs structures is always a challenge.

To address above challenge, we attempted to utilize contrastive pre-learning strategies to alleviate medical image tasks with small annotated datasets and serious deviations in the distribution of cross-institutional data, to strengthen model feature extraction capability. Then we propose a parallel multiscale progressive refinement graph neural network (PCG-Net) for segment HN OARs, which contains A parallel encoder (PE), a cascade graph module (CGM), and a progressive refinement module (PRM). In addition, we proposed a new loss function based on the combination of dice scores and focal losses, for better segmenting small OARs structures.

To evaluate the performance of PCG-Net, we conducted experiments using two publicly available datasets and two local datasets for HN OARs segmentation. We performed a systematic analysis of various components of PCG-Net and compared them with other segmentation methods to demonstrate the effectiveness of PCG-Net’s components. Furthermore, we utilized three distinct downstream tasks to evaluate the robustness of PCG-Net. The evaluation of PCG-Net indicating its potential for HNC treatment and various clinical applications.

## 2 Related works

### 2.1 Siamese-contrastive learning

**Figure 1:**
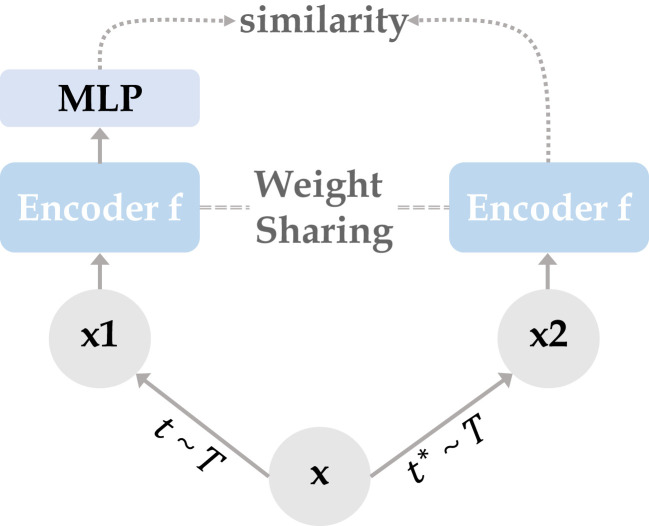
Two mutually independent augmentation operators *t*, $t^{*}$ are randomly sampled from the data augmentations cluster *T*, and applied to the input x to obtain two correlated views x1, x2. Two identical encoder f (CNN/Transformer + MLP) are trained using x1 and x2, then the predicted MLP is applied on one side while the other side stops the gradient update, using the negative cosine similarity to minimize the feature difference of the two output results. After completing the training, we remove all MLP layers and use CNN or Transformer encoder for downstream tasks.


The overall architecture of Siamese contrastive learning is shown in **Figure 1**. Two randomly augmented feature maps $x_{1}$ and $x_{2}$ from the input image x are fed to the encoder f , which includes a backbone network (CNN or Transformer) and a multi-layer perceptron MLP for performing prediction functions. The two output vectors are denoted as $z_{i}≜P_{r}(f(x_{i}))$ and $p_{i}≜f(x_{i})$ , where i represents the input number and $P_{r}$ represents MLP for performing prediction. The difference between $p_{i}$ and $z_{i}$ is minimized with negative cosine similarity as in equation (1), with optimizing encoder f by Siamese-loss function as in equation (2), where ||·||_2_ is $\ell_{2}$ -norm, S is the stop-gradient operation. S specifically presents as $S(p_{1},\;stopgrad(z_{2}))$ , and $S(p_{2},stopgrad(z_{1}))$ , which means the gradient of $z_{i}$ is replaced by constant. Therefore, equation (2) can be updated to equation (3), expressed as the encoded network on $x_{1}$ receiving the back-propagation gradient from $p_{1}$ in the first term, while receiving no back-propagation gradient from $z_{1}$ in the second term (and vice versa for $x_{2}$ ).

(1) $$\begin{array}{c} S(p_{1},z_{2})=-\frac{p_{1}}{∥p_{1}{∥}_{2}}\cdot \frac{z_{2}}{∥z_{2}{∥}_{2}}\\ S(p_{2},z_{1})=-\frac{p_{2}}{∥p_{2}{∥}_{2}}\cdot \frac{z_{1}}{∥z_{1}{∥}_{2}} \end{array}$$

(2) $$L=\frac{1}{2}S\left(\left(p_{1},z_{2}\right)\right)+\frac{1}{2}S\left(\left(p_{2},z_{1}\right)\right)$$

(3) $$L=\frac{1}{2}S\left((,p_{1}\text{,stopgrad }(\left(z_{2},)\right),)\right)+\frac{1}{2}S\left((,p_{2},\text{stopgrad }\left((,z_{1},)\right),)\right)$$

## 3 Method

**Figure 2:**
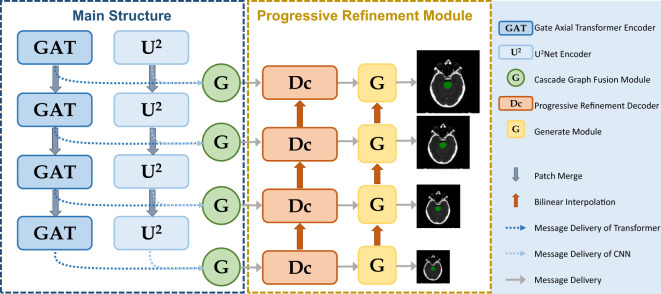
PCG-Net extracts local features and global contextual information through a parallel encoder, progressive refinement architecture for resolution-by-resolution spatial information recovery to achieve efficient feature decoding, cascade graph modules embedded in skip-connections adaptively refine high-level representations between different semantic information to achieve feature fusion and transfer. (The meanings of different modules represented in the figure are given in the legend).

The parallel multiscale progressive refinement graph neural network PCG-Net based on Siamese-contrastive learning is shown in **Figure 2**. PCG-Net uses parallel encoder (PE) and cascade graph module (CGM) to extract and fusion local features and global contextual information, respectively (Further details are available in the **Supplementary Materials**). In addition, the prediction results are progressively refined from lower resolution to higher resolution by the progressive refinement module (PRM) to optimize segmentation details.

### 3.1 Parallel encoder

#### 3.1.1 Gated-axial transformer encoder

**Figure 3:**
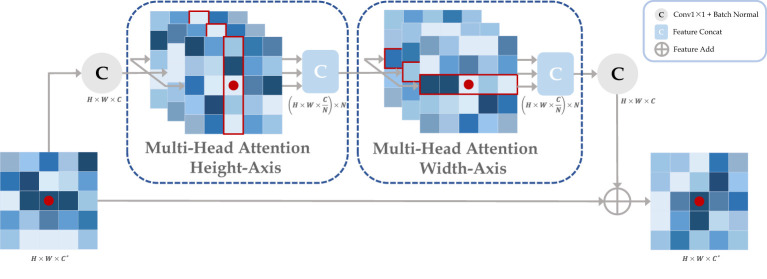
The axial attention module is composed by cascading the attention layers in height and width axis. Taking the red point as an example, it performs a multi-headed self-attention calculation with other points along specific columns and specific rows in sequence. Ultimately, the red point features contain all the information about the row and column in which it is located. where H, W, C, and N represent the height, width, channel, and attention head of the feature map, respectively, and ***C*** * represents the original input channel.


In this work, the traditional self-attention layers were replaced by two axial modules, which performed self-attention operations on the height-axis and width-axis, respectively, as shown in **Figure 3**. Specifically, the 2D spatial operation of the traditional self-attention layer was transformed into a 1D axial operation, and self-attention encoding was performed for the height and width axes sequentially. A multiple-headed attention mechanism was employed for both axis modules to optimize encoding performance. In addition, we define three positional bias matrices $r_{ij}^{q}$ , $r_{ij}^{k}$ , $r_{ij}^{v}\in R^{W\times W}$ to encode positional parameters $q_{ij}$ , $k_{ij}$ , $v_{ij}$ to accurately capture more accurate positional information, respectively, where $q_{ij}$ , $k_{ij}$ , $v_{ij}$ , represent the query, key and value, respectively. These bias matrices can participate in the gradient descent of neural networks to update the weights parameters. Finally, we introduced the gate mechanism to adaptively control the effect of the position bias on the output $y_{ij}$ during the self-attention encoding process, the width-axis self-attention operations is shown in equation (4), where $G_{Q},G_{K},G_{V1},G_{V2}\in R$ are learnable parameters which forms the gate bias.

(4) $$y_{ij}(x_{ij})=\sum_{w=1}^{W}\text{softmax}\left(q_{iw}^{T}k_{iw}+G_{Q}q_{iw}^{T}r_{iw}^{q}+G_{K}k_{iw}^{T}r_{iw}^{k}\right)\left(G_{V1}v_{iw}+G_{V2}r_{iw}^{v}\right)$$

#### 3.1.2 CNN encoder

Although the transformer architecture enables sufficient extraction of global information, due to the self-attention mechanism, the transformer is prone to ignore local details. Without excellent feature extraction capability from the local to global, the organ contours cannot be accurately segmented. To extract both local features and global context information, the CNN encoder was utilized to compensate for the deficiencies of the transformer encoder. The U-shaped architecture has already been widely used in medical artificial intelligence, which usually builds U-shaped cascades based on sequential stacking of VGG architectures. But it has been demonstrated that single-level U-shaped architectures are susceptible to losing semantic details in deeper networks (21). Therefore, an $U^{n}Net$ encoder was introduced to alleviate the gradient loss problem, where n could be set as any positive integer to achieve multi-level or single-level nesting. Here, we set n as 2 to build the $U^{2}Net$ encoder. Its exterior is semi-U-shaped with top-down compression of spatial information into channel information. Each module internally is independently U-shaped nested, which can effectively extract intra-stage multi-scale features and aggregate inter-stage multi-level features.

### 3.2 Cascade graph module

**Figure 4:**
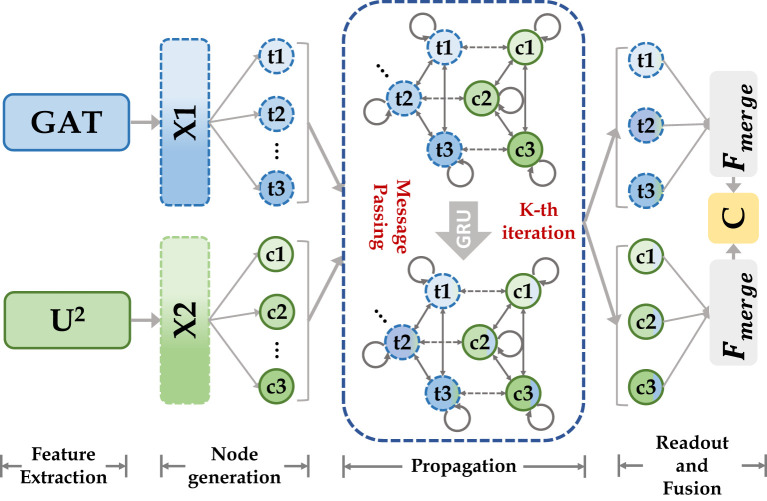
Our cascade graph module is built on two different categories of feature extractors (CNN and Transformer). Node features are updated by message-passing functions and gated recurrent neural networks, they enable inference of high-level relationships between different semantic space nodes and construct more powerful feature representations.


We use CGM for fusing high-level semantic information extracted based on the transformer encoder and CNN encoder, as shown in **Figure 4**. We first define two types of nodes: global feature nodes $V_{1}=\{t_{1},t_{2},\ldots ,t_{n}\}$ and local feature nodes $V_{2}=\{c_{1},c_{2},\ldots ,c_{n}\}$ , where n represents the nodes number, $t_{i}$ and $c_{i}$ represents feature node. their initial feature scales are both $t_{i}^{\left(0\right)},c_{i}^{\left(0\right)}\in R^{c\times h\times w}$ , where c,h,w are the number of node channels, height, and width, respectively. For capturing feature information at different receptive fields, 2n nodes are obtained by using n different dilated convolutions with different dilated rates applied to two different types of feature maps. The integral node encoding can be represented as equation (5). where $\delta^{m}$ denotes the dilated convolution, m denotes the dilated rate, and $\chi_{h\times w}$ ensures the spatial dimension of the feature map after interpolation is h×w .

(5) $$\begin{array}{l} t_{i}^{\left(0\right)}=\chi_{h\times w}(Conv_{\delta }^{m}(X_{1};\delta^{m}))\\ c_{i}^{\left(0\right)}=\chi_{h\times w}(Conv_{\delta }^{m}(X_{2};\delta^{m})) \end{array}$$

Then we defined two types of edge $e_{1}$ and $e_{2}$ to update node state. For $e_{1}$ , the relationship function is represented as equation (6), where ϑ is defined as the aggregation function between the same type of nodes, which can be represented by $\vartheta (t_{i}^{\;},t_{j}^{\;})=\alpha t_{i}^{\;}+\beta t_{j}^{\;}$ , where i and j denote the node numbers. The connection relation $t_{i}↔c_{i}$ was defined as $e_{2}$ , which can be expressed by the function $f_{\xi }$ , with the overall process shown in equation (7), where α,β∈R is the learnable gating unit.


(6) $$e_{1}=Conv\{\sum_{i=1}^{n}\sum_{j=1}^{n}\vartheta [(t_{i},t_{j})or(c_{i},c_{j})]\}\in R^{c\times h\times w}$$

(7) $$e_{2}=f_{\xi }(V_{1},V_{2})=Conv[\sum_{i=1}^{n}\sum_{j=1}^{n}\left(\alpha t_{i}+\beta c_{j}\right)]\in R^{c\times h\times w}$$

Finally, we defined two types message passing function *M* (same feature node aggregation $M_{1}$ and different feature node aggregation $M_{2}$ ) for aggregating information from neighboring nodes to update the central node, as shown in equation (8), where δ is the sigmoid function. Gated Recurrent Unit (GRU) was used to update the node state as shown in equation (9). After the t message passing steps, every node in nodes set contains the feature information from neighboring nodes to achieve the effect of feature fusion. Finally, all the updated nodes were merged to generate dense mapping of the feature map, as shown in equation (10), where $F_{merge}$ is the 3 × 3 convolutional layer and $X^{\Theta }$ is the output after GNN feature fusion.


(8) $$\begin{array}{l} M_{1}^{\left(t-1\right)}=\sum_{i=1}^{2}V_{i}[\delta (e_{1}^{\left(t-1\right)})]\\ M_{2}^{\left(t-1\right)}=V[\delta (e_{2}^{\left(t-1\right)})] \end{array}$$

(9) $$V^{\left(t\right)}=F_{GRU}(V^{\left(t-1\right)},M^{\left(t-1\right)})$$

(10) $$X^{\Theta }=Conv_{C^{\kappa }}(F_{merge}(V_{1},V_{2}))\in R^{C^{\kappa }\times h\times w}$$

### 3.3 Progressive refinement module

The decoder contains a series of up-sampling modules to gradually recover spatial information. For each decoding block, the feature map scale resolution increases by a factor of 2 and skip-connects with the output of CGM, which not only introduces multi-dimensional spatial information but also alleviates the common gradient problem in deep learning. Usually, low resolution compared to high resolution makes reconstruction easier and focuses more on global features (22). Therefore, PRM was introduced to gradually add detailed information during decoding to generate more accurate predictions. Specifically, each prediction branch of the decoding module contains a generator G to generate target region contours $\varphi_{i}^{G}$ of scale $n_{i}\times n_{i}$ . Each generator consists of two successive series $\gamma_{i}$ (convolution, batch normalization, ReLu activation function) and a feature dimension-adjusted convolution $\sigma_{i}$ . The successive series $\gamma_{i}$ at low resolution, after bilinear interpolation up-sampling with the scale factor of 2, are fed to the higher scale prediction branch to perform elementwise addition with the output of successive series $\gamma_{i+1}$ at higher resolution, and the targets’ contours $\varphi_{i+1}^{G}$ at the current scale resolution are obtained by dimension-adjusted convolution $\sigma_{i+1}$ . The overall progressive refinement branch is shown in equation (11), where ⊕ is the elementwise addition and U is the up-sampling operation.


(11) $$\varphi_{i}^{G}=\{\begin{array}{ll} \sigma_{i}{\left(\gamma \right)}_{i}, & i=1\\ \sigma_{i+1}(\gamma_{i+1}⊕U(\gamma_{i})), & i=2,3,4 \end{array}$$

### 3.4 Loss function

The number of voxels within the small target volume is considerably fewer than the number of voxels outside, which means the data distribution is unbalanced and could lead to difficulty in training. Therefore, small target segmentation has always been a challenge in semantic segmentation. To address the above issues, the loss function fusion algorithm was employed to make the model fit target volume contours more accurately. The dice loss (23) enables converting the voxels-by-voxels labeling problem into minimizing the class-level distribution distance, which can alleviate the shortcoming in small target volume contributing slightly to the loss function. The focal loss (24) is the extension based on the cross-entropy loss function, which can adaptively apply different weights to distinct voxels to further alleviate the problems of difficulty imbalance in segmentation. In PCG-Net, the dice loss $l_{DSC}$ was used to reduce the imbalance voxel problem, focal loss $l_{Focal}$ was used to strengthen the model to focus on misclassified voxels, in order to design and build the focal-dice loss function $l_{DF}$ , as shown in equation (12), where $FP_{p}(m)$ , $FN_{p}(m)$ and $TP_{p}(m)$ are the false positives, false negatives and true positives of class m based on the predicted probabilities, respectively. $p_{n}(m)$ is the predicted probability that voxel n belongs to class m, and $g_{n}(m)$ is the ground truth that voxel n belongs to class m, where m is the total number of OARs structures plus one (background), and n is the total number of voxels in the CT image. α=2 is a weight parameter to balance between $l_{DSC}$ and $l_{Focal}$ . β=1 and η=1 are the trade-offs of penalties for false negatives and false positives.


(12) $$\begin{array}{l} TP_{p}(m)=\sum_{n=1}^{N}p_{n}(m)g_{n}(m)\\ FN_{p}(m)=\sum_{n=1}^{N}g_{n}(m)(1-p_{n}(m))\\ FP_{p}(m)=\sum_{n=1}^{N}(1-g_{n}(m))p_{n}(m)\\ l_{DF}=l_{DSC}+\alpha l_{Focal}\\ \;=M-\sum_{m=0}^{m-1}\frac{TP_{p}(m)}{TP_{p}(m)+\beta FN_{p}(m)+\eta FP_{p}(m)}\\ \;-\alpha \frac{1}{N}\sum_{m=0}^{m-1}\sum_{n=1}^{N}g_{n}(m){\left(1-p_{n}(m)\right)}^{2}\log(p_{n}(m)) \end{array}$$

## 4 Experiment

### 4.1 Dataset

In the HN OARs segmentation task, our data include two publicly available datasets: DATASET 1 (177 samples) consisting of CT images from four different institutions in Quebec, Canada, and DATASET 2 (46 samples) consisting of CT images from the Head-Neck Cetuximab collection, as well as two local datasets: DATASET 3 (60 samples) provided by the Department of Radiology, Hubei Cancer Hospital, and DATASET 4 (100 samples) provided by the Radiotherapy Center of Anhui Provincial Hospital. Each dataset contains five organs: brain stem, mandible, parotid, optic chiasm, and optic nerve. Please note that for detailed information on publicly available datasets (DATASET 1 and DATASET 2), please refer to reference (25). The explanations about the acquisition of local CT datasets (DATASET 3 and DATASET 4) are as follows: During CT simulation, patients were immobilized in supine position with a thermoplastic mask and underwent contrast-enhanced CT scan on the CT scanning system (Philips Brilliance Big Bore, GE LightSpeed 16, and GE Discovery CT590 RT). The resolution, and thickness of CT images were 512 × 512× (0.9766-1.1719mm), and 2.5mm-3 mm, respectively.

The two publicly available datasets contain CT images from five different institutions, which have significant data complexity. Therefore, during the contrastive experiments shown in Section 5.3, contrastive learning was performed based on the public dataset (223 samples) to pre-train the encoder for improving the robustness and feature extraction capability of the encoder. DATASET 4 was used as the training dataset for supervised learning to fine-tune the weight distribution of the neural network. DATASET 3 was used to validate and test the effectiveness of the algorithm. In particular, the pre-training process requires only CT images without corresponding manually delineation, whereas the training and validation processes both require HN CT images and corresponding manually delineated OARs. It was ensured that the above four datasets are not overlapping with each other to avoid any potential overfitting.

To demonstrate the heterogeneity between the public dataset and the local dataset, the following features were extracted from each image using the gray-level co-occurrence matrix: sum entropy, difference entropy, sum average, correlation, contrast, homogeneity, sum variance, and variance, then the statistical differences were analyzed between the datasets. The p-value of each statistic was then obtained using the Mann-Whitney U test. The results in **Table 1** show that eight statistics have p-values less than 0.001, hence there are serious adaptation issues between the two types of datasets which require more powerful pre-training methods with segmentation algorithms to adapt to both datasets.

**Table 1:** Analysis of the statistical differences.

Textural Features	p-value	Textural Features	p-value
Sum entropy	< 0.001	Difference entropy	< 0.001
Sum average	< 0.001	Correlation	< 0.001
Contrast	< 0.001	Homogeneity	< 0.001
Sum variance	< 0.001	Variance	< 0.001

To further test our algorithm’s efficacy in different downstream tasks, more datasets were collected, including 1) Liver Tumor Segmentation Challenge (LITS) liver cancer public dataset, with 131 patient samples, 2) Lung Nodule Analysis 16 (LUNA16) lung cancer public dataset, with 888 patient samples, and 3) (Blood Cell Classification Datasets) BCCD blood cell classification public dataset, containing 12,500 blood cell enhanced images (JPEG format).

### 4.2 Evaluation metrics

The segmentation performance was evaluated by calculating the Dice Similarity coefficient (DSC), which is defined as $DSC(p,z)=\frac{2\times |p\cap z|}{\left|p|+|z\right|}\times 100\%$ , where p is the voxel mask predicted by the network and z is the ground truth. The DSC values are between 0 and 1, where the closer DSC is to 1, the better the segmentation performance. In addition, to evaluate the segmentation results from multiple perspectives, the Hausdorff Distance (HD), the Average Symmetric Surface Distance (ASSD), and the Jaccard Coefficient (Jaccard) were further utilized as supplementary metrics. Generally, the DSC and Jaccard are considered more sensitive to the voxel details inside the contour which reflects the segmentation integrity, while the HD and ASSD are more sensitive to the contour surface which can characterize the segmentation surface contour accuracy. The four-evaluation metrics complement each other and enable a comprehensive assessment of segmentation results. Please note that the p-value of each statistic in our work was derived by other methods with PCG-Net based on the T-test: Two-tailed critical value for paired sample mean analysis.

### 4.3 Experimental details

The neural network using PyTorch was implemented and experiments were performed on a small NVIDIA RTX3090Ti workstation equipped with 24GB of RAM. To enhance data consistency and improve model training efficiency, all CT images and mask labels were preprocessed in the same way. Using the linear interpolation method to adjust the pixel spacing of different institutions’ images, each slice pixel spacing was adjusted to 1mm, and the original CT images and the masked images were padded to 512×512 uniformly. Image morphing is to rotate, translate, mirror, and affine transform each CT image with its corresponding label to enhance the complexity of the data. The grayscale float uses the current voxel grayscale value superimposed with random initialization numbers, which in turn generates CT images with noise, thus effectively improving the model’s anti-interference capability. Please refer to the **Supplementary Material** for more details on about image preprocessing and grayscale float.

For the contrastive learning pre-trained encoder, the SGD optimizer was used for pre-training. Linear scaling learning rate (26) was used with a base $l_{r}=0.05$ , and the learning rate has a cosine decay schedule (27). The weight decay was 0.0001 and the SGD momentum was 0.9. Considering the computational complexity, the batch size was set to 32 and the epoch size was set to 50. For training PCG-NET, Adam with a weight decay of 0.0001 was utilized to optimize network parameters, with the initial learning rate set to 0.0001, and the ‘‘ploy’’ strategy with 0.9 power as adjustment. The batch size was set to 32 and the epoch size was set to 150 due to hardware limitations.

## 5 Results

To demonstrate the benefits brought by each module and the superiority of PCG-Net, the following experiments were performed: the benefits of gated-axial transformer encoder, cascade graph feature fusion architecture, and progressive refinement decoder on PCG-Net through ablation study was demonstrated in Section 5.1; the superiority of PCG-Net’s was verified by comparing it with three advanced segmentation algorithms, U^2^Net, CPFNet, and MedT in Section 5.2; the effectiveness of Siamese contrastive learning pre-trained encoder was demonstrated in Section 5.3; the universality and generalization ability of PCG-Net by other medical tasks was demonstrated in Section 5.4.

It’s worth noting that in Sections 5.1 and Sections 5.2, the contrastive learning strategy was not utilized to pre-train the PCG-Net’s encoder, while the overall training method was the supervised task, with DATASET 4 as the training dataset, and DATASET 3 as the validation and test dataset. In Section 5.3, in order to discuss the importance of contrastive learning, the encoder was first pre-trained by DATASET 1 and DATASET 2, which was an unsupervised task. Secondly, the pre-trained encoder weights were transferred to PCG-Net, during which the MLP layer necessary for the contrastive learning task was removed, and end-to-end training of the PCG-Net by DATASET 4 was performed based on a supervised strategy, with DATASET 3 as the validation and testing dataset. Please note that all the results are the mean values on the test datasets after ten-fold cross-validation. In addition, the detailed processing times for all deep learning models handling the same image can be found in the **Supplementary Materials**.

### 5.1 Ablation study

To demonstrate the effectiveness of different modules, ablation experiments were performed to compare the gains from each module. Using the $U^{2}Net$ (28) as a baseline, unlike traditional $U^{2}Net$ which contains 6 encoder/decoder blocks, a 4 encoder/decoder blocks structure was employed to reduce the computational complexity. For better performance, the pooling layer was replaced by the patch merging layer (29) for minimizing the semantic information loss caused by the traditional pooling layer. During the ablation study, all competitors were conducted in the same computing environment and under the same data enhancement to ensure a fair comparison.

**Figure 5:**
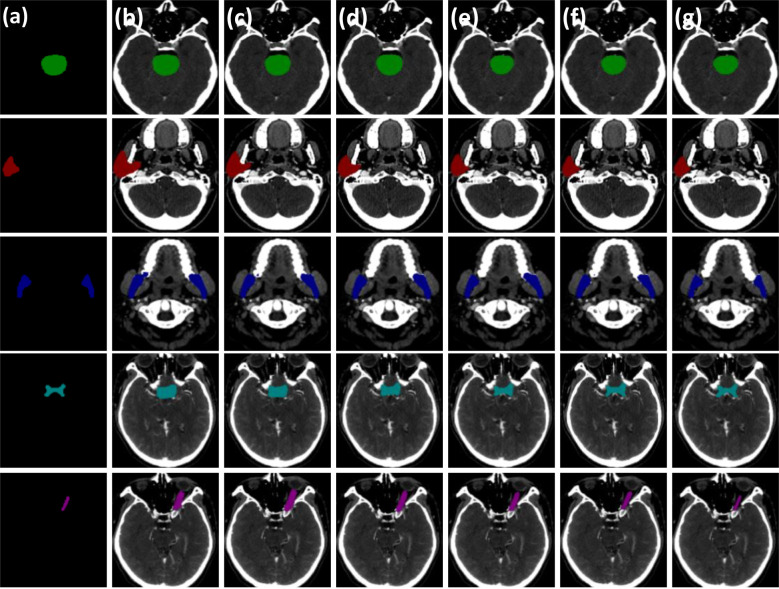
Visual comparison of three critical modules of the PCG-Net ablation study. **(A)** Ground truth. **(B)** Baseline model. **(C)** The progressive refinement decoder replaces the baseline decoder. **(D)** Gated-axial transformer encoder replaces the baseline encoder. **(E)** The parallel encoder replaces the baseline encoder. **(F)** The parallel encoder replaces the baseline encoder and cascade graph module replaces the baseline skip connection. **(G)** The parallel encoder replaces the baseline encoder, the progressive refinement decoder replaces the baseline decoder, and the cascade graph module replaces the baseline skip connection. To facilitate visual representation, we used different color masks to represent different organ-at-risks, where green mask is the brainstem, red mask is the parotid, blue mask is the mandible, cyan mask is the optic chiasm, and purple mask is the optic nerve.

By replacing the corresponding components in the baseline network with the gated-axial transformer, the cascaded graph module, and the progressive refinement decoder, respectively, it was possible to obtain: level1 (progressive refinement decoder replacing the baseline decoder), level2 (gated-axial transformer encoder replacing the baseline encoder), and level3 (parallel encoder replacing the baseline encoder). Further, the following was obtained by simultaneous replacement for two or three components in the baseline network: level4 (parallel encoder replacing the baseline encoder, cascade graph module replacing the baseline skip connection), level5 (parallel encoder replacing the baseline encoder, progressive refinement decoder replacing the baseline decoder, cascade graph module replacing the baseline skip connection). Five methods equipped with different modules were evaluated on the HN dataset, with the segmentation results shown in **Figure 5**. Compared with the baseline method, the level1, level2, and level3 methods have improvements in processing segmentation tasks. Compared to adding only a single module to the baseline, the combination based on two or more modules can obtain more accurate segmentation results, especially for small volume OARs. The statistical results are shown in **Table 2**.

**Table 2:** Statistical comparisons of ablation studies for the three main modules in PCG-Net.

	Brain Stem	Mandible	Parotid	Optic Nerve	Optic Chiasm	Mean	Mean (small volume OARs)
**Baseline**	84.3% ± 2.6%	85.2% ± 3.1%	78.8% ± 3.7%	65.5% ± 7.8%	52.2% ± 14.3%	73.2% ± 6.3%	58.85% ± 11.05%
	77.2% ± 2.1%	76.3% ± 2.7%	74.4% ± 3.1%	58.2% ± 6.9%	44.1% ± 10.2%	66.0% ± 5%	51.15% ± 8.55%
	3.123 ± 1.1	3.211 ± 0.9	4.212 ± 1.7	2.979 ± 0.6	4.811 ± 1.2	3.67 ± 1.1	3.895 ± 0.9
	1.217 ± 0.4	1.334 ± 0.5	1.899 ± 0.7	0.886 ± 0.3	1.577 ± 0.6	1.38 ± 0.5	1.2315 ± 0.45
**Level 1**	85.1% ± 2.1%	85.9% ± 2.2%	79.3% ± 3.1%	66.1% ± 7.3%	53.1% ± 12.9%	73.90% ± 5.5%	59.6% ± 10.1%
	77.9% ± 1.8%	78.1% ± 2.3%	74.7% ± 3.3%	58.8% ± 7.1%	45.7% ± 11.4%	67.04% ± 5.2%	52.25% ± 9.25%
	3.013 ± 1.3	3.313 ± 1.2	3.991 ± 1.9	2.876 ± 0.9	4.792 ± 1.3	3.60 ± 1.3	3.834 ± 1.1
	1.155 ± 0.4	1.270 ± 0.4	1.792 ± 0.8	0.878 ± 0.3	1.565 ± 0.6	1.33 ± 0.5	1.221 ± 0.45
**Level 2**	84.9% ± 2.3%	85.8% ± 2.4%	80.1% ± 3.2%	65.9% ± 7.1%	49.4% ± 16.3%	73.22% ± 6.3%	57.65% ± 11.7%
	78.2% ± 2.0%	78.7% ± 2.5%	75.1% ± 2.9%	54.3% ± 9.2%	41.3% ± 14.2%	65.52% ± 6.2%	47.8% ± 11.7%
	2.992 ± 0.9	3.172 ± 0.9	3.876 ± 1.5	2.878 ± 0.9	5.137 ± 1.9	3.61 ± 1.22	4.008 ± 1.4
	1.143 ± 0.3	1.233 ± 0.5	1.786 ± 0.7	0.924 ± 0.4	1.669 ± 0.9	1.35 ± 0.56	1.296 ± 0.65
**Level 3**	87.1% ± 2.6%	88.7% ± 1.9%	85.9% ± 2.7%	68.8% ± 6.7%	57.1% ± 9.2%	77.52% ± 4.6%	62.95% ± 7.95%
	81.8% ± 2.3%	82.5% ± 1.9%	78.8% ± 3.0%	60.2% ± 5.5%	48.0% ± 8.3%	70.26% ± 4.2%	54.1% ± 6.9%
	2.633 ± 1.2	3.017 ± 1.3	3.663 ± 1.6	2.531 ± 0.5	4.331 ± 1.1	3.24 ± 1.1	3.431 ± 0.8
	0.983 ± 0.3	1.005 ± 0.5	1.594 ± 0.5	0.775 ± 0.2	1.347 ± 0.4	1.14 ± 0.4	1.061 ± 0.3
**Level 4**	88.9% ± 1.9%	90.7% ± 2.1%	87.9% ± 2.9%	70.2% ± 6.5%	57.7% ± 10.7%	79.08% ± 4.8%	63.95% ± 8.6%
	82.2% ± 2.2%	83.3% ± 2.3%	79.2% ± 2.7%	61.9% ± 6.1%	48.8% ± 9.1%	71.08% ± 4.5%	55.35% ± 7.6%
	2.455 ± 0.8	2.967 ± 0.9	3.532 ± 1.3	2.411 ± 0.6	4.299 ± 1.3	3.13 ± 1.0	3.355 ± 0.95
	0.916 ± 0.4	1.005 ± 0.4	1.511 ± 0.6	0.701 ± 0.2	1.210 ± 0.5	1.07 ± 0.4	0.955 ± 0.35
**Level 5**	89.2% ± 1.9%	91.8% ± 2.1%	89.1% ± 2.6%	71.7% ± 6.9%	58.1% ± 12.1%	79.98% ± 5.1%	64.9% ± 9.5%
	82.9% ± 1.9%	84.1% ± 2.0%	79.7% ± 2.5%	63.1% ± 5.7%	49.1% ± 7.9%	71.78% ± 4.0%	56.1% ± 6.8%
	2.406 ± 0.9	2.655 ± 1.1	3.317 ± 1.4	2.389 ± 0.5	4.221 ± 1.1	3.00 ± 1	3.305 ± 0.8
	0.881 ± 0.3	0.993 ± 0.3	1.470 ± 0.6	0.677 ± 0.2	1.112 ± 0.4	1.03 ± 0.4	0.894 ± 0.3

In each ablation experiment, the first and second rows represent the DSC values (mean ± variance) and Jaccard values (mean ± variance) in the test datasets, respectively; the third and fourth rows represent the HD_(mm)_ values (mean ± variance) and ASSD_(mm)_ values (mean ± variance) in the test datasets, respectively. The results in the table are the mean values on the test datasets after ten-fold cross-validation.

Compared with the baseline network, the mean DSC of the level1 and level2 methods improved by approximately 0.7% (from 73.2% to 73.9%) and 0.02% (from 73.2% to 73.22%), respectively, which proved contribution of the progressive refinement module and the gated-axial transformer module in feature decoding and feature encoding. Compared with the baseline model and level2, the mean DSC and mean Jaccard of the level3 method improved by 4.32% (from 73.2% to 77.52%), 4.26% (from 66.0% to 70.26%) and 4.3% (from 73.22% to 77.52%), 4.74% (from 65.52% to 70.26%), respectively, while the mean DSC and mean Jaccard of the small volume OARs (optic chiasm and optic nerve) improved by 4.1% (from 58.85% to 62.95%), 5.3% (from 57.65% to 62.95%) and 2.95% (from 51.15% to 54.1%), 6.3% (from 47.8% to 54.1%), respectively, demonstrating that the parallel encoder is superior to the single-branch encoder in segmentation accuracy, which enables adequate extraction of the local information while fitting the global information. Compared with the level3 method, the segmentation accuracy for the level4 method further improved, with the mean DSC and mean Jaccard improving by 1.56% (from 77.52% to 79.08%) and 0.82% (from 70.26% to 71.08%), respectively. For the small volume OARs (optic chiasm and optic nerve), the mean DSC and mean Jaccard improved by 1.00% (from 62.95% to 63.95%) and 1.25% (from 54.1% to 55.35%), respectively, revealing the excellent feature fusion and relationship modeling capabilities of the cascade graph module. The level5 method, simultaneously integrating three modules, achieved the best global prediction results, with the mean DSC and mean Jaccard improving by 6.78% (from 73.2% to 79.98%) and 5.78% (from 66.0% to 71.78%) compared to the baseline model. In the small volume OARs segmentation (optic chiasm and optic nerve), the improvement was particularly significant compared with the baseline model, with the mean DSC and mean Jaccard for optic chiasm improving by 6.05% (from 58.85% to 64.9%) and 4.95% (from 51.15% to 56.1%).

### 5.2 Model horizontal comparison

PCG-Net was horizontally compared with three other advanced segmentation approaches, including U^2^Net (28), CPFNet (30), and MedT (31). In the comparison experiments, all competitors were performing under the same computational environment and the same data enhancement to ensure a fair comparison. **Table 3** depicts the segmentation results by different methods on the HN OARs. Our model achieved the most excellent results on most metrics, with mean DSC, mean Jaccard, mean HD, and mean ASSD of 79.98%, 71.78%, 3.00, and 1.03, respectively. On small volume OARs segmentation, compared to the MedT, the mean DSC, mean Recall, mean HD, and mean ASD of our model improved by 1.08%, 1.5%, 3.23%, and 1.90%, respectively.

**Table 3:** Statistical comparison with different state-of-the-art methods.

	Brain Stem	Mandible	Parotid	Optic Nerve	Optic Chiasm	Mean
**U^2^Net(×)**	84.3% ± 2.6%^*^	85.2% ± 3.1%^*^	78.8% ± 3.7%^**^	65.5% ± 7.8%^*^	52.2% ± 14.3%^*^	73.20% ± 6.30%^*^
	77.2% ± 2.1%^**^	76.3% ± 2.7%^*^	74.4% ± 3.1%^*^	58.2% ± 6.9%^#^	44.1% ± 10.2%^***^	66.04% ± 5.00%^#^
	3.123 ± 1.1^*^	3.211 ± 0.9^*^	4.212 ± 1.7^**^	2.979 ± 0.6^*^	4.811 ± 1.2^*^	3.67 ± 1.10^*^
	1.217 ± 0.4^*^	1.334 ± 0.5^**^	1.899 ± 0.7^***^	0.886 ± 0.3^*^	1.577 ± 0.6^*^	1.38 ± 0.50^#^
**CPFNet(×)**	88.1% ± 2.4%^**^	88.7% ± 2.7%^#^	85.1% ± 2.9%^**^	70.3% ± 7.1%^*^	56.2% ± 13.3%^*^	77.68% ± 5.68%^**^
	80.5% ± 2.1%^*^	83.1% ± 2.3%^*^	77.2% ± 3.7%^*^	61.1% ± 6.2%^*^	47.8% ± 8.0%^***^	69.94% ± 4.46%^*^
	2.662 ± 0.8^*^	2.932 ± 0.9^**^	3.636 ± 1.5^*^	**2.377 ± 0.6** ^**^	4.667 ± 1.1^*^	3.25 ± 0.98^**^
	1.003 ± 0.3^*^	1.113 ± 0.3^*^	1.517 ± 0.6^**^	0.689 ± 0.2^**^	1.225 ± 0.4^*^	1.11 ± 0.36^#^
**MedT(×)**	**89.5% ± 1.7%** ^*^	90.6% ± 1.9%^***^	88.6% ± 3.1%^*^	68.1% ± 8.8%^*^	57.7% ± 12.9%^*^	78.90% ± 5.68%^***^
	82.2% ± 2.1%^*^	83.7% ± 2.2%^*^	78.3% ± 2.9%^*^	59.2% ± 6.7%^*^	48.0% ± 7.5%^*^	70.28% ± 4.28%^*^
	2.513 ± 0.9^*^	2.717 ± 1.1^#^	3.379 ± 1.4^*^	2.511 ± 0.5^*^	4.375 ± 1.2^**^	3.10 ± 1.02^**^
	**0.879 ± 0.2** ^*^	1.059 ± 0.4^*^	**1.447 ± 0.5** ^*^	0.703 ± 0.4^*^	1.169 ± 0.3^*^	1.05 ± 0.36^*^
**PCG-Net** **(×)**	89.2% ± 1.9%	**91.8% ± 2.1%**	**89.1% ± 2.6%**	**71.7% ± 6.9%**	**58.1% ± 12.1%**	79.98% ± 5.12%
	**82.9% ± 1.9%**	**84.1% ± 2.0%**	**79.7% ± 2.5%**	**63.1% ± 5.7%**	**49.1% ± 7.9%**	71.78% ± 4.00%
	**2.406 ± 0.9**	**2.655 ± 1.1**	**3.317 ± 1.4**	2.389 ± 0.5	**4.221 ± 1.1**	3.00 ± 1.00
	0.881 ± 0.3	**0.993 ± 0.3**	1.470 ± 0.6	**0.677 ± 0.2**	**1.112 ± 0.4**	1.03 ± 0.36
**U^2^Net(√)**	84.7% ± 2.3%^*^ 77.9% ± 1.6%^*^	85.6% ± 3.0%^*^	79.3% ± 3.5%^*^	66.7% ± 6.8%^*^	53.7% ± 11.9%^*^	74.00% ± 5.50%^*^
		76.9% ± 2.5%^*^	74.9% ± 3.2%^*^	59.5% ± 5.4%^***^	45.3% ± 8.8%^*^	66.90% ± 4.30%^***^
	3.112 ± 1.2^*^	3.157 ± 0.7^*^	4.106 ± 1.5^**^	2.858 ± 0.5^*^	4.551 ± 1.1^*^	3.56 ± 1.00^**^
	1.179 ± 0.3^*^	1.298 ± 0.4^*^	1.847 ± 0.7^*^	0.831 ± 0.2^**^	1.436 ± 0.3^*^	1.32 ± 0.38^*^
**CPFNet(√)**	88.3% ± 2.2%^*^	89.1% ± 2.7%^**^	85.8% ± 2.3%^*^	71.2% ± 6.7%^*^	58.3% ± 10.7%^#^	78.54% ± 4.92%^*^
	81.2% ± 2.0%^*^	83.6% ± 2.3%^#^	77.9% ± 3.5%^*^	63.3% ± 6.1%^*^	48.2% ± 8.2%^#^	70.84% ± 4.42%^*^
	2.636 ± 0.8^*^	2.919 ± 0.7^*^	3.596 ± 1.2^*^	2.290 ± 0.5^**^	4.544 ± 0.9^*^	3.20 ± 0.82^**^
	0.997 ± 0.2^**^	1.107 ± 0.3^*^	1.403 ± 0.5^*^	0.676 ± 0.2^*^	1.193 ± 0.3^*^	1.08 ± 0.30^#^
**MedT(√)**	89.9% ± 1.6%^*^	91.2% ± 1.8%^*^	89.1% ± 2.7%^**^	69.4% ± 7.9%^*^	58.6% ± 10.9%^*^	79.64% ± 4.98%^**^
	82.7% ± 2.0%^*^	84.2% ± 1.9%^*^	78.9% ± 2.6%^*^	60.1% ± 6.8%^*^	49.3% ± 8.1%^**^	71.04% ± 4.28%^*^
	2.479 ± 0.8^#^	2.699 ± 1.0^*^	3.293 ± 1.5^*^	2.410 ± 0.7^#^	4.132 ± 1.5^*^	3.00 ± 1.10^**^
	0.873 ± 0.3^*^	1.016 ± 0.3^*^	1.431 ± 0.4^***^	0.688 ± 0.3^*^	1.027 ± 0.4^*^	1.01 ± 0.34^*^
**PCG-Net** **(√)**	***90.1% ± 2.2%***	***92.3% ± 1.9%***	***89.9% ± 2.4%***	***73.2% ± 7.3%***	***59.9% ± 11.3%***	81.08% ± 5.02%
	***83.3% ± 1.7%***	***84.7% ± 1.8%***	***80.2% ± 2.1%***	***64.6% ± 6.1%***	***50.7% ± 7.2%***	72.70% ± 3.78%
	***2.377 ± 0.8***	***2.613 ± 1.0***	***3.288 ± 1.1***	***2.157 ± 0.4***	***4.023 ± 1.3***	2.89 ± 0.92
	***0.868 ± 0.3***	***0.986 ± 0.3***	***1.436 ± 0.5***	***0.619 ± 0.3***	***1.013 ± 0.3***	0.98 ± 0.34

In each comparison experiment, the first and second rows represent the DSC values (mean ± variance) and Jaccard values (mean ± variance) in the test datasets, respectively; the third and fourth rows represent the HD _(mm)_ values (mean ± variance) and ASSD _(mm)_ values (mean ± variance) in the test datasets, respectively. The symbols at the bottom of the models in the first column represent with/without contrastive pre-training strategy, where (×) indicates without contrastive learning pre-training strategy and (√) indicates with contrastive learning pre-training strategy. The black bold font indicates the optimal value among the four models without the contrastive learning pre-training strategy. The black bold italic font indicates the optimal value among the four models with the contrastive learning pretraining strategy. Please note that “***” to indicate p< 0.05, “**” for p< 0.01, “*” for p< 0.001, and “#” for p > 0.05.

**Figure 6:**
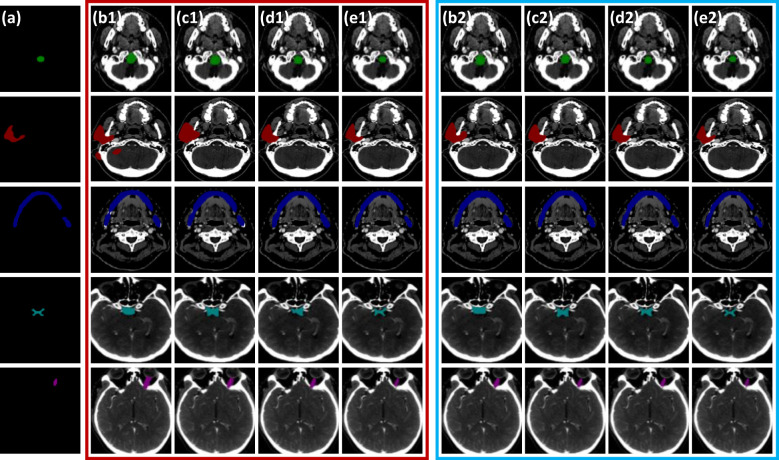
Horizontal visual comparison of PCG-Net with different state-of-the-art algorithms. Where the red box represents the segmentation result without a contrastive learning pre-training strategy, and the blue box represents the segmentation result with a contrastive learning pre-training strategy. **(A)** Ground truth. **(B)** U^2^Net. **(C)** CPFNet. **(D)** MedT. **(E)** PCG-Net. To facilitate visual representation, we used different color masks to represent different organ-at-risks, where green mask is the brainstem, red mask is the parotid, blue mask is the mandible, cyan mask is the optic chiasm, and purple mask is the optic nerve.

For visual comparison, the results of different segmentation algorithms are shown in **Figure 6**. Significant superiority can be observed for our algorithm compared to other competitors, especially for the more accurate identification of the small volume OARs. Combining **Figure 6** with **Table 3**, PCG-Net effectively extracted local features and global context information by parallel encoder, fused features by cascaded module, and used progressive refinement decoder gradually refines the spatial dimension. Therefore, our segmentation accuracy was superior compared with other competitors, especially in the case of small volume OARs and blurred foreground and background boundaries.

### 5.3 Contrastive learning evaluation

**Figure 7:**
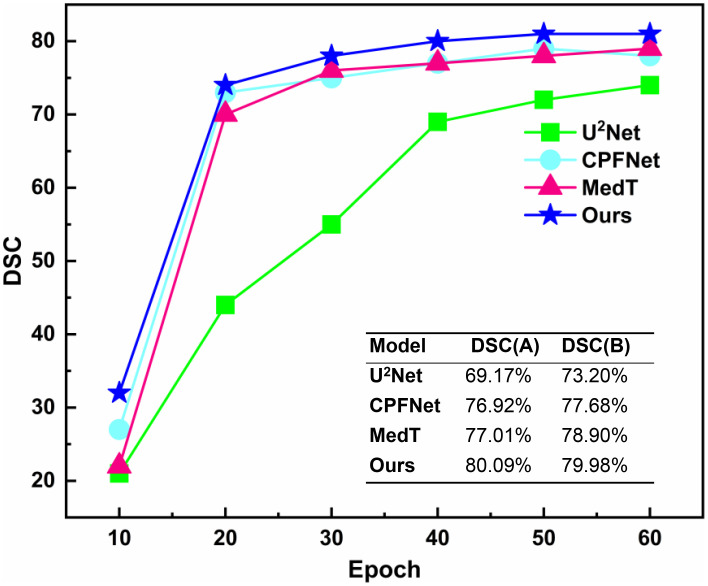
Mean DSC versus epoch for automatic head and neck organ-at-risks segmentation of the test datasets by different neural network models based on contrastive learning strategy. DSC(A) represents the mean DSC value of the head and neck organs-at-risks in the test datasets when different models were trained to the 40_th epoch under the contrastive pre-training strategy. DSC(B) represents the mean DSC values of the head and neck organ-at-risks in the test datasets when different models were trained to the end (150_th epochs) without the contrastive pre-training strategy.

The unsupervised strategy of contrastive learning was applied to the current main segmentation algorithms, including U^2^Net (28), CPFNet (30), and MedT (31). All competitors were performing under the same computing environment and the same data enhancement throughout the experiment to ensure fair comparisons. The experimental algorithms were classified into two categories: one using contrastive learning pre-training strategy and the other without contrastive learning pre-training strategy. The overall results of the comparison of the gain of the four different main segmentation algorithms with difference in whether contrastive learning was imposed are shown in **Table 3**. After applying the contrastive learning strategy, the models showed slightly improved segmentation accuracy for large volume OARs. For example, for the brainstem and mandible, the mean DSC of the four models improved by 0.45% and 0.475%, respectively. The segmentation accuracy significantly improved for small volume OARs. For example, for optic nerve and optic chiasm, the mean DSC of the four models improved by 1.225% and 1.575% after using contrastive learning, respectively. This may be because the unsupervised paradigm of contrastive learning enables effective extraction of the similar features from large amounts of data to improve the neural network’s weight distribution. The mean DSC values versus epoch for different OARs based on contrastive learning strategies using supervised tasks to fine-tune the four neural network weights is plotted in **Figure 7**. The accuracy of each algorithm reached the optimal value of the contrastive-free learning strategy after about 40 epochs. This fully demonstrated the feasibility of using contrastive learning to perform unsupervised training on large medical unlabeled samples and transferring the pre-trained model to supervised tasks for weight fine-tuning. This strategy greatly solved the problem of medical tasks with few annotated data. The experiment results further verified that contrastive learning has a strong generalization ability, which can find common-solution in distinct datasets with statistically significant differences to optimize feature extraction module weights. With the epoch gradually increasing, the accuracy of MedT and U^2^Net gradually stabilizes, while the accuracy of CPFNet slightly decreases, which is probably caused by model overfitting. Compared with the competitors, the accuracy of PCG-Net has been steadily improving, and its mean DSC always remains at the highest level, which fully verifies the advanced performance of PCG-Net.

### 5.4 Other medical assignments

The contrastive-learning-based PCG-Net achieved excellent results on the HN segmentation task, which can not only accurately delineate large volume OARs such as parotid gland and brainstem, but also accurately identify small volume samples such as optic chiasm and optic nerve. However, the HN segmentation tasks alone cannot fully demonstrate the superiority of PCG-Net. Therefore, three different medical image challenge tasks were chosen, including segmentation, classification, and object detection, to further validate the generalization ability of PCG-Net. For different downstream tasks, different decoders were used while ensuring the feature extraction module remaining constant. For example, in classification tasks, the progressive refinement decoder was replaced by the fully connected layer, and the final output was the mapping of category numbers. To perform the object detection task, the progressive refinement decoder was replaced by the YOLOV3 decoder, and output was in three different scales of detection windows to achieve object detection for different sizes. All experiments were performed in the same computing environment and data enhancement to ensure fairness.

#### 5.4.1 Liver tumor segmentation

A horizontal comparison experiment for liver tumor segmentation was performed on the LiTS dataset, where the training, validation, and testing sets were divided with the ratio of 7:2:1, using SGD optimizer with momentum of 0.9 and linear scaling learning rate with weight decay of 0.0001, with focal-dice loss as loss function, batch size set to 32, epoch set to 150, using DSC, VOE, and ASSD as evaluation metrics. The LiTS dataset includes primary and secondary liver tumors with strong heterogeneity and diffuseness. Therefore, it can be fully verified whether the algorithm can effectively extract features from the region of interest to achieve end-to-end mapping under the circumstances of blurred boundaries, complex structure, diverse distribution, and grayscale diversity.

**Figure 8:**
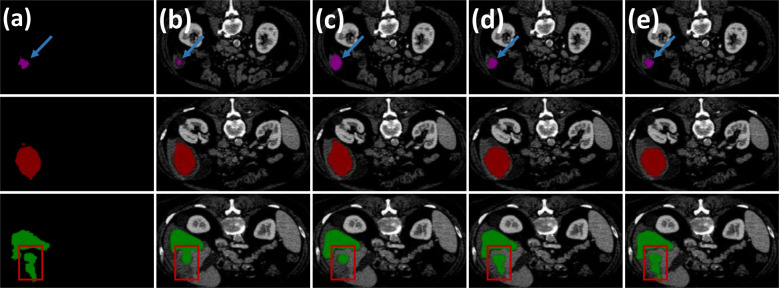
Visual comparison of PCG-Net on the LiTS dataset with different state-of-the-art methods. **(A)** Ground truth. **(B)** U^2^Net. **(C)** SFF-Net. **(D)** H-Dense UNet. **(E)** PCG-Net. Three different types of tumors were selected to fully demonstrate PCG-Net’s effectiveness: small tumors (purple mask), large tumors (red mask), and multiple tumors (green mask). The blue arrow highlights PCG-Net’s ability to accurately identify the irregular boundary contours of small tumors compared to other algorithms, and the red box highlights PCG-Net’s ability to correctly capture multiple tumors.

PCG-Net was compared with four currently popular segmentation methods, including SFF-Net (32), H-Dense UNet (33), and FAT-Net (34). The segmentation results of applying different algorithms on the LiTS dataset are shown in **Table 4**, including accuracy and computational complexity evaluation metrics. The SFF-Net based on a multi-scale feature pyramid and feature fusion module obtained relatively accurate results for the large tumor (diameter larger than 10 mm) segmentation problem. However, the performance was unsatisfactory for the segmentation of small tumors (diameter less than 5 mm) and multiple tumors. The H-Dense UNet, which relies on the fusion of 2D features with 3D features to increase the spatial region of interest, achieved comparable performance to the FAT-Net method. However, both methods underperformed in edge-complex multi-tumor semantic segmentation problems. On the contrary, PCG-Net based on a parallel encoder, cascade graph module, and progressive refinement decoder can effectively reconstruct the dependencies between different features and achieve end-to-end mapping by layer-by-layer refinement. PCG-Net performed better than other competitors in general, with the highest mean DSC of 73.6%, and mean VOE and mean ASSD metrics of 21.19% and 1.118, respectively. Meanwhile, the parametric number of PCG-Net was lower than average, indicating PCG-Net reduced the computational complexity without losing segmentation accuracy. In addition, the visualization of comparison of segmentation results between different algorithms is demonstrated in **Figure 8**. For most samples with extremely complex blurred boundaries and diverse grayscales, PCG-Net still obtained the best segmentation results.

**Table 4:** Horizontal comparison experiment of liver tumor segmentation based on Lits dataset, where ↑ indicates the larger value the better, and ↓ indicates the smaller value the better.

	DSC (%)↑	VOE (%)↓	ASSD (mm) ↓
**SFF-Net**	61.3% ± 11.8%^*^	39.8% ± 14.3%^**^	1.885 ± 0.5^*^
**H-Dense UNet**	71.3% ± 10.9%^**^	25.7% ± 13.6%^#^	1.331 ± 0.3^*^
**FAT-Net**	72.3% ± 15.2%^**^	24.26% ± 14.1%^***^	1.371 ± 0.2^*^
**PCG-Net**	**73.6% ± 7.8%**	**21.19% ± 9.7%**	**1.118 ± 0.2**

Please note that “***” to indicate p< 0.05, “**” for p< 0.01, “*” for p< 0.001, and “#” for p > 0.05.

The bold values denote the optimal result.

#### 5.4.2 Lung nodule object detection

A horizontal comparison experiment for lung nodule object detection was performed on the LUNA16 dataset, where the training, validation, and testing sets were divided in 7:2:1, using an SGD optimizer with a momentum of 0.9 and linear scaling learning rate with weight decay of 0.0001, using EIoU as the loss function (35), with batch size set to 32 and epoch size set to 150. The model performance was evaluated by CPM competitive performance metrics. The LUNA16 lung nodule dataset is challenging for object detection because of the extremely complex brightness distribution and blurred boundaries. The YOLOV3 encoder was replaced with the PCG-Net encoder, Att-UNet encoder (36), U2Net encoder (28), ViT encoder (37), and Swin (29) encoder, respectively, to demonstrate the feature extraction capability of the PCG-Net encoder. The results of object detection applying different encoders on the LUNA16 dataset, including the sensitivity to the seven average number of false positives per scan and the corresponding computational complexity evaluation metrics, are shown in **Table 5**. The U2Net, which introduced U-shaped nested architecture with multi-level and multi-scale feature extraction modules, achieved more accurate object recognition results than AttU-Net. The ViT based on long-distance feature modeling had significantly higher computational complexity and slightly lower detection accuracy compared to the traditional feature extraction modules AttU-Net and U2Net. Swin alleviated the high computational complexity of ViT through the shift-window mechanism, hierarchical structure, and window self-attention. However, the results in **Table 5** demonstrate that Swin still performed poorly on few-sample medical object detection tasks. PCG-Net can effectively solve the latter three problems by trainable positional bias and gated-axial transformer encoder, and alleviate the first problems by the parallel encoder. Therefore, through the parallel encoder and cascaded graph neural networks, it is capable to accurately capture and model local features and global context information, meanwhile reducing overfitting risks. It is observed from **Table 5** that our algorithm performed better than other competitors, with the highest mean CPM at 92.0%.

**Table 5:** Horizontal comparison experiment for lung nodule object detection based on the LUNA16 dataset.

Model	Number of FPs/scan (%)							CPM(%)↑
	A (%)	B (%)	C (%)	D (%)	E (%)	F (%)	G (%)	
**Att-UNet Encoder**	71.2%	81.1%	85.7%	88.6%	91.2%	93.3%	94.1%	86.5%
**U^2^Net Encoder**	68.8%	72.6%	81.3%	86.5%	89.9%	93.0%	94.7%	83.8%
**ViT Encoder**	78.8%	87.3%	91.7%	92.2%	92.9%	93.6%	94.5%	90.1%
**Swin Encoder**	79.5%	87.8%	92.1%	92.6%	93.1%	93.7%	94.6%	90.4%
**PCG-Net Encoder**	**80.3%**	**87.9%**	**90.2%**	**93.2%**	**96.2%**	**97.7%**	**98.5%**	**92.0%**

In the table, A, B, C, D, E, F, and G represent the sensitivity of detection at an average number of false positives of 0.125, 0.25, 0.5, 1, 2, 4, and 8 in each group of CT images, respectively. For a fair comparison, we deploy the opponent’s model locally, and train them employing the same preprocessing, optimizer, loss function, and training epochs, where ↑ indicates that the larger value the better.

The bold values denote the optimal result.

#### 5.4.3 Classification of blood cells

A horizontal comparison experiment for blood cell classification was performed on the BCCD dataset. The BCCD dataset includes four different types of cells: neutrophils, monocytes, lymphocytes, and eosinophils. Blood disease diagnosis usually involves classifying blood cell subtypes, having very important clinical significance. A four-classified fully connected layer was used as decoder, using ResNet50 (38), RepLKNet (39), ViT (36), and ConvNeXt (40) as encoder to verify the feature extraction capability of PCG-Net. Because of the few samples of the BCCD dataset, it is prone to overfitting when the BCCD dataset was directly used for training. Therefore, contrastive learning was employed to pre-train the encoder of five different models on the LIDC-IDRI dataset, then the BCCD dataset was used to fine-tune encoder weights. The training, validation, and testing sets were divided in the ratio of 7:2:1, using an SGD optimizer with a momentum of 0.9 and linear scaling learning rate with a weight decay of 0.0001, using cross entropy as the loss function, with batch size set to 32 and epoch size set to 150. The results of the blood cell classification task applying different encoders on the BCCD dataset are shown in **Table 6**, including accuracy and computational complexity evaluation metrics. Using large convolutional kernels enabled RepLKNet and ConvNeXt to increase the receptive field more efficiently than ResNet50, while avoiding optimization difficulties caused by the increase of model depth, therefore, the classification accuracy was significantly improved. However, large convolution kernels increased the computational complexity, which cannot balance accuracy and speed. The ViT, which is the classical classification architecture of the transformer, had mediocre performance, with no improvement in classification accuracy despite the increase in computational complexity. Our structure utilized small convolutional kernels of CNN to efficiently extract local semantic information, gated axial self-attention architecture to reduce computational complexity without losing receptive fields, and cascaded graph module as feature fusion architecture to achieve efficient information aggregation. It can be observed in **Table 6** that PCG-Net achieved the highest scores under most evaluation metrics, especially the two major metrics, Accuracy and Spec, which reached 98.6% and 99.0%, respectively.

**Table 6:** Horizontal comparison experiment of blood cell classification based on BCCD dataset.

	ACC(%)↑	Spec(%)↑	Sens(%)↑
**ResNet50**	93.6% ± 1.9%^*^	79.3% ± 6.3%^*^	98.8% ± 1.3%^**^
**RepLKNet**	98.0% ± 0.8%^***^	97.1% ± 2.3%^**^	**98.4% ± 1.3%** ^*^
**ViT**	94.3% ± 1.7%^*^	96.6% ± 5.5%^*^	92.1% ± 1.1%^**^
**ConvNext**	98.3% ± 0.7%^#^	98.3% ± 0.9%^*^	98.2% ± 1.2%^*^
**PCG-Net**	**98.6% ± 0.3%**	**99.0% ± 1.0%**	98.3% ± 0.7%

For a fair comparison, we deploy the opponent’s model locally, and train them employing the same preprocessing, optimizer, loss function, and training epochs, where ↑ indicates that the larger value the better. Please note that “***” to indicate p< 0.05, “**” for p< 0.01, “*” for p< 0.001, and “#” for p > 0.05.

The bold values denote the optimal result.

## 6 Discussion

In this study, a Siamese-contrastive learning strategy was used to pre-train encoder weights on public datasets and transfer them to local tasks for fine-tuning. It can be seen from the results that this pre-training strategy can be used to fit local tasks by using prior knowledge of public datasets, which can be crucial in the case of sparse annotation samples. The essence of deep learning is extraction and generalization for large amounts of features. Annotated samples enable the model to extract more reliable information from datasets, however medical data require manual annotation by numerous professional physicians, which is extremely costly. Therefore, learning efficient visual representation without annotated samples is the focus of the medical task. Currently, the unsupervised tasks were mainly based on generative or contrastive learning. Generative learning, represented by self-encoders (41), generates or models pixels in the input space (42), yet the pixel-level generation consumes considerable computational resources. The contrastive learning method (43) uses the loss function similar to supervised tasks to optimize the weight distribution, which can autonomously learn the mapping relationships among large amounts of data and ignore the complex details of instances, therefore the optimization of model becomes simpler (44).

Encoder-decoder architectures have been widely used in medical artificial intelligence tasks, but most algorithms used a single type codec to extract features, such as UNet (45) for pure CNN architectures and MedT (31) for pure transformer architectures, thereby not being able to simultaneously capture local features and global contextual information. Fused CNN and transformer architectures such as Confomer (46) and CoTNet (47) are difficult to be applied directly in local datasets, although they have achieved state-of-the-art results in their fields, both requiring pre-training with large amounts of data to fit the neural network. Other parallel encoding algorithms such as FAT-Net (34) have achieved state-of-the-art results in dermatological segmentation. However, FAT-Net does not employ an effective feature fusion method, only stacking the CNN’s and transformer’s high-dimensional semantic features at the bottom of the encoder, which ignores the importance of features under parallel encoders of different scales. In summary, those methods still failed to effectively fuse local features and global long-term dependencies. Furthermore, traditional transformer algorithms require enormous data, making it difficult for direct application to medical few-sample tasks. Therefore, most available methods still failed to accurately infer the small volume OARs of the lesion areas with blurred boundaries. To obtain better feature extraction capability, balance local information with global context information, and applicability to few-sample datasets, we integrated CNN and axial transformer branches for a parallel encoder, making local and global information supplement each other to achieve accurate feature extraction, where the transformer branch adopts cascaded axial architecture, which can alleviate the computational complexity (48) without losing spatially distant features, and effectively solve the problems of heavy expenses and resource consumption of traditional self-attention mechanism. The advantages were clearly demonstrated by the ablation studies and comparison experiments conducted in Section 5.1.

Although abundant local features and global contextual information were extracted by a parallel encoder, accurate target segmentation is impossible if they cannot be aggregated by an effective message-passing method. Therefore, a cascaded graph neural network model was used to refine the high-level relationship between two different feature spaces to improve the model representation. Extensive work have been done to improve segmentation performance by fusing semantic information under different feature spaces. For instance, DCA (49) directly stacked and spliced two features to improve the semantic representation, and FSSD (50) extracted various scale features from a different layer of the model for contact. However, these methods only focused on information transfer and ignored modeling and reasoning between different features, which makes them difficult to fully utilize features of different spatial resolutions or different semantic categories to overcome complex medical tasks. Numerous experiments have demonstrated GNNs to be sensitive to relational modeling and feature inference (51–53), so the cascaded graph neural network model used in this study enabled aggregating different feature information by learning powerful and dense feature embeddings. It is proved that this cascade graph model can capture detailed regions and overcome ambiguities by employing the complementary information of multi-level features.

Finally, a novel parallel multiscale progressive refinement graph neural network PCG-Net was proposed to achieve accurate OARs segmentation in the presence of unbalanced data and few annotated samples to assist physicians clinically. To evaluate the contribution of each module to the PCG-Net, ablation studies were performed for each module to demonstrate their effectiveness. Comparing with the advanced segmentation algorithms U^2^Net (28), SFF-Net (32), H-Dense UNet (33), and FAT-Net (34), PCG-Net showed stronger feature extraction capability and robustness. To verify PCG-Net’s fitting ability for different tasks, the encoder of PCG-Net was applied to the three main vision downstream tasks respectively. In the context of head and neck segmentation tasks, when compared to the commonly used medical image segmentation algorithm, MedT, PCG-Net demonstrates notable improvements in the segmentation of small organs, specifically the optic nerve and optic chiasm. PCG-Net achieves a 1.08% increase in DSC, a 1.5% improvement in Recall, a 1.5% reduction in HD, and a 1.9% decrease in ASD. Furthermore, in various downstream medical image tasks, PCG-Net consistently delivers outstanding results. For instance, in the context of liver cancer segmentation, PCG-Net outperforms all listed models, achieving a DSC, VOE, and ASSD of 73.6%, 21.19%, and 1.118, respectively. In comparison to SSF-Net, PCG-Net exhibits substantial improvements of 16%, 46.7%, and 40.6%, significantly enhancing liver cancer recognition capabilities. The results proved that PCG-Net had strong generalization ability for different tasks. Note that the encoder of PCG-Net can be used as a backbone feature extraction module for different medical tasks in different datasets. Meanwhile, the pre-training approach based on contrastive learning can effectively overcome the weakness of insufficient annotated data in medical tasks, and this may be the priority method for processing medical tasks in the future.

The PCG-Net proposed in this study still has limitations. Similar to most existing neural network models, PCG-Net can only be trained for specific tasks due to local computing power and algorithmic constraints. In practical applications, it requires pre-trained models with different data to handle different downstream tasks, which greatly increases resource consumption and workload. In addition, contrastive learning can significantly reduce the amount of annotated data required by neural networks, however, during the model training process, it still requires fine-tuning model weights with annotated samples to fit the ground truth, which cannot completely achieve unsupervised training. In the future work, we will focus on the study of model generalization and unsupervised tasks.

## 7 Conclusion

A PCG-Net was proposed to solve the problems of few clinical medical images, lack of annotated data, and difficulty in segmenting small volume OARs. Using the contrastive learning pre-training strategy, the local task was fitted by prior knowledge from large unannotated datasets, which greatly alleviates the model robustness problem caused by sample scarcity. Unlike traditional single-branch encoders, our parallel encoder can infer semantic features from two different dimensions, effectively extracting global contextual information while preserving local receptive fields. In addition, the cascade graph architecture could allow better utilization of abundant complementary information in multi-level features compared to traditional fusion methods. Extensive experiments were conducted to evaluate PCG-Net on different medical tasks and compare it horizontally with the current main approaches in different downstream tasks, further demonstrating the excellent inference performance and generalization capability of PCG-Net. It is believed that the novel design in this paper could be effectively used for clinical applications and treatment.

## Data availability statement

The raw data supporting the conclusions of this article will be made available by the authors, without undue reservation.

## Ethics statement

Written informed consent was obtained from the individual(s) for the publication of any potentially identifiable images or data included in this article.

## Author contributions

SL: Conceptualization, Methodology, Writing – original draft, Writing –review and editing. CW: Conceptualization, Methodology, Writing – original draft, Writing –review and editing. YD: Conceptualization, Methodology, Writing – original draft, Writing –review and editing. XX: Conceptualization, Methodology, Funding acquisition, Writing –review and editing. WW: Conceptualization, Funding acquisition, Project administration, Methodology, Writing – original draft, Writing –review and editing. XY: Methodology. XW: Methodology, Writing –review and editing. CM: Methodology, Writing –review and editing. BZ: Conceptualization, Methodology, Writing – original draft, Writing –review and editing, Project administration.
