# Supplementary material for: PCG-net: feature adaptive deep learning for automated head and neck organs-at-risk segmentation
Source: Front Oncol. 2023 Oct 20;13:1177788. doi: 10.3389/fonc.2023.1177788 (PMC10623055; doi:10.3389/fonc.2023.1177788)
Supplement: Supplementary file 1 [file DataSheet_1.pdf]

Supplementary Table 1. The detailed processing times for all deep learning models handling the same image can be found in the supplementary materials.

Case	U^2Net	CPF-Net	Med-T	SFF-Net	H-Dense UNet	FAT-Net	PCG-Net
(128,128)	~0.0276 sec	~0.0358 sec	~0.0329 sec	~0.0316 sec	~0.0557 sec	~0.0863 sec	~0.0303 sec

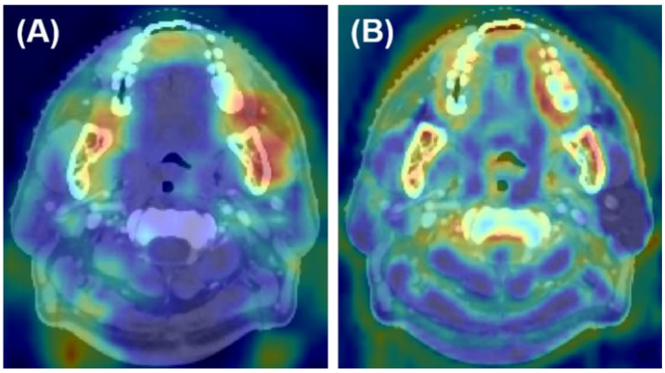

Supplementary Figure 1. The attention maps of the first layer of the parallel encoder using the head and neck CT images.

The Grayscale Float Strategy.

The grayscale float $G^f(x, y, z)$, which introduces noise into the CT images, can be mathematically represented as follows:

$$G^f(x,y,z) = G(x,y,z) + N * R(x,y,z)$$

Here's what each component represents:

$G^f(x, y, z)$: The resulting grayscale value after floating, introducing noise into the original grayscale value $G(x, y, z)$.

$G(x, y, z)$: The original voxel grayscale value at coordinates (x, y, z).

$R(x, y, z)$: A random initialization value at coordinates (x, y, z). These random initialization values are drawn from a specified probability distribution.

N: A scaling factor that controls the intensity of noise. By adjusting the value of N, you can control the overall noise level in the generated CT images. A higher N increases the noise intensity, while a lower N reduces it.

Additionally, the type of noise and its characteristics depend on the probability distribution from which $R(x, y, z)$ is drawn. For example, using a Gaussian distribution or other distributions can result in different noise patterns and textures in

the CT images, allowing you to manipulate the noise type. The size of the random initialization matrix or vector can also be adjusted to control the spatial extent of noise in the CT images. These parameters, including N and the choice of distribution for $R(x, y, z)$, are tuned through empirical testing and domain-specific knowledge to achieve the desired level of noise and enhance the model's resilience against interference.

Data Preprocessing Workflow.

Initially, we conducted preprocessing on hospital DICOM format CT data, converting them into array formats. we adhered to a preprocessing workflow for training neural networks, which encompassed the following strategies: **Data Acquisition:** We acquired the raw CT image data stored in DICOM format. **Data Quality Control:** We meticulously screened the acquired data, conducting stringent quality control checks to identify artifacts, noise, or any other anomalies. In instances where data exhibited irregularities, we performed manual corrections or, if necessary, excluded the data from analysis. **Data Format Conversion:** The original data, initially stored in DICOM or alternative formats, underwent conversion into image formats amenable to analysis, such as the NIfTI (Neuroimaging Informatics Technology Initiative) format. **Image Resampling:** CT images obtained from different medical devices or institutions may have varying pixel spacing, we resampled them to a uniform spatial resolution and size. This standardization facilitated seamless neural network analysis. **Intensity Normalization:** To ensure uniformity of image intensities across data originating from diverse scanning devices or collected at different time points, we employed intensity normalization. This measure served to prevent any potential analytical ambiguities. **Noise Reduction and Smoothing:** Recognizing the presence of motion artifacts within patient CT data, we applied noise reduction and smoothing techniques. These interventions effectively mitigated image noise and improved overall image quality. **Region of Interest (ROI) Extraction:** Depending on the specific research objectives, we delineated head and neck organs -at-risks, such as Brain Stem, Mandible, Parotid, Optic Chiasm, Optic Nerves, as the

designated region of interest (ROI). This selection served to define the scope of our analysis with precision. **Data Storage:** Lastly, the preprocessed data and extracted features were securely stored for subsequent stages of analysis and modeling.
